# Safety, Biodistribution, and Radiation Dosimetry of ^18^F-rhPSMA-7.3 in Healthy Adult Volunteers

**DOI:** 10.2967/jnumed.120.252114

**Published:** 2021-05-10

**Authors:** Tuula Tolvanen, Kari Kalliokoski, Simona Malaspina, Anna Kuisma, Salla Lahdenpohja, Ernst J. Postema, Matthew P. Miller, Mika Scheinin

**Affiliations:** 1 Turku PET Centre, University of Turku and Turku University Hospital, Turku, Finland; 2 Department of Oncology and Radiotherapy, Turku University Hospital, Turku, Finland; 3 Blue Earth Diagnostics Ltd., Oxford, United Kingdom; and; 4 Clinical Research Services Turku–CRST Ltd., Turku, Finland

**Keywords:** ^18^F, biodistribution, dosimetry, PSMA, rhPSMA

## Abstract

This first-in-humans study investigated the safety, biodistribution, and radiation dosimetry of a novel ^18^F-labeled radiohybrid prostate-specific membrane antigen (rhPSMA) PET imaging agent, ^18^F-rhPSMA-7.3. **Methods:** Six healthy volunteers (3 men, 3 women) underwent multiple whole-body PET acquisitions at scheduled time points up to 248 min after the administration of ^18^F-rhPSMA-7.3 (mean activity, 220; range, 210–228 MBq). PET scans were conducted in 3 separate sessions, and subjects were encouraged to void between sessions. Blood and urine samples were collected for up to 4 h after injection to assess metabolite-corrected radioactivity in whole blood, plasma, and urine. Quantitative measurements of ^18^F radioactivity in volumes of interest over target organs were determined directly from the PET images at 8 time points, and normalized time–activity concentration curves were generated. These normalized cumulated activities were then inputted into the OLINDA/EXM package to calculate the internal radiation dosimetry and the subjects’ effective dose. **Results:** ^18^F-rhPSMA-7.3 was well tolerated. One adverse event (mild headache, not requiring medication) was considered possibly related to ^18^F-rhPSMA-7.3. The calculated effective dose was 0.0141 mSv/MBq when using a 3.5-h voiding interval. The organs with the highest mean absorbed dose per unit of administered radioactivity were the adrenals (0.1835 mSv/MBq), the kidneys (0.1722 mSv/MBq), the submandibular glands (0.1479 mSv), and the parotid glands (0.1137 mSv/MBq). At the end of the first scanning session (mean time, 111 min after injection), an average of 7.2% (range, 4.4%–9.0%) of the injected radioactivity of ^18^F-rhPSMA-7.3 was excreted into urine. **Conclusion:** The safety, biodistribution, and internal radiation dosimetry of ^18^F-rhPSMA-7.3 are considered favorable for PET imaging.

Prostate-specific membrane antigen (PSMA) is a transmembrane enzyme that is overexpressed in prostate cancer cells compared with healthy tissue (1). Its extracellular catalytic site allows targeting with specific small-molecule inhibitors or antibodies that may subsequently become internalized (2). PET radiopharmaceuticals such as Glu-NH-CO-NH-Lys-(Ahx)-[^68^Ga]-HBED (^68^Ga-PSMA-11) use this ligand binding for the purpose of prostate cancer imaging, particularly in patients with biochemical recurrence (3). However, some characteristics of ^68^Ga-PSMA-11, such as its rapid excretion into urine, causing substantial accumulation in the urinary bladder, can be a particular disadvantage for pelvic imaging in patients with prostate cancer (4,5).

^18^F-labeled PSMA agents are increasingly used in preference to ^68^Ga-labeled ones because of the favorable characteristics of the ^18^F isotope. These include a longer half-life, capability for production of larger batches, a higher positron yield, and lower positron energy, which results in decreased image noise and improved contrast resolution compared with ^68^Ga-labeled counterparts (6,7). Many ^18^F-labeled PSMA ligands have been used clinically, in particular 2-(3-{1-carboxy-5-[(6-^18^F-fluoro-pyridine-3-carbonyl)-amino]-pentyl}-ureido)-pentanedioic acid (^18^F-DCFPyL) and ((3*S,*10*S,*14S)-1-(4-(((*S*)-4-carboxy-2-((*S*)-4-carboxy-2-(6-^18^F-fluoronicotinamido)butanamido)butanamido)methyl)phenyl)-3-(naphthalen-2-ylmethyl)-1,4,12-trioxo-2,5,11,13-tetraazahexadecane-10,14,16-tricarboxylic acid) (^18^F-PSMA-1007) have been used in large numbers of patients (8). The former has relatively high urinary excretion, whereas the latter has very low urinary excretion, since it is eliminated mainly via the bile (9). Although the imaging properties of ^18^F-DCFPyL, ^18^F-PSMA-1007, ^68^Ga-PSMA-11, and several other diagnostic PSMA tracers have been well described and all have their individual strengths, none of these radiopharmaceuticals are being used to treat patients. The current portfolio of therapeutic PSMA ligands consists of compounds such as ^177^Lu PSMA-617 (vipivotide tetraxetan Lu-177), PSMA I&T (Glu-CO-Lys[(Sub)DLys-DPhe-DTyr(3I)-DOTAGA] trifluoroacetate), and PSMA-R2 (Nε-[^177^Lu(4,7,10-Tricarboxymethyl-1,4,7,10-tetrazacyclododec-1-yl)acetyl]-6-Aminohexanoic)-(Nε′-4-Bromobenzyl) Lysine-CO-Glutamic-acid, but no true theranostic pair is currently available.

Radiohybrid PSMA (rhPSMA) ligands are a new class of compounds that can be efficiently labeled with ^18^F or with radioactive metal isotopes and, consequently, offer diagnostic and therapeutic PSMA targeting. ^18^F-rhPSMA-7, which comprises 4 diastereoisomers, has shown promising preliminary imaging characteristics in patients with prostate cancer (10,11), and Gallium 2,2′,2″-(10-((3S,7S,12R,26R,34S)-1,3,7,12,26,34-hexacarboxy-29- ((4-(di-tert-buty[^18^F]fluorosilyl)benzamido)methyl)-5,10,17,20,28,31-hexaoxo- 4,6,11,16,21,27,30-heptaazatetratriacontan-34-yl)-1,4,6,10- tetraazacyclododecane-1,4,7-triyl)triacetate, ^18^F-rhPSMA-7.3, is now under clinical development, seeking formal approval for registration in the United States and in the European Union.

Here, we present results of a phase 1, open-label study designed to evaluate the safety, biodistribution, and internal radiation dosimetry of ^18^F-rhPSMA-7.3 in healthy adult volunteers. Quantification of the in vivo activity at multiple times after administration is fundamental in determining the biodistribution of the radionuclide of interest. In almost all cases in the development of a diagnostic radiopharmaceutical (12–17), the biodistribution is initially measured in healthy volunteers, largely because of the impracticality of having patients undergo whole-body imaging at several acquisition time points, which are required for a complete assessment of biodistribution (18). Imaging times can be long and patient compliance (e.g., in terms of remaining still) may be difficult to attain if the patient is in physical discomfort. In the present study design, the guidance provided by the European Medicines Agency was considered—the biodistribution in healthy volunteers can be considered normal, given the strict entry criteria regarding the patient’s health, concomitant medication, and lifestyle (i.e., smoking and use of alcohol and drugs), to minimize the risk for confounding factors (19). In keeping with European practice, the estimated effective dose to the healthy volunteers should not exceed 10 mSv (risk category IIb (20)). Since uptake of PSMA tracers in other tumors has been described in the literature (21), the group of healthy volunteers should not be limited to male volunteers but should also include female individuals to reflect the normal biodistribution of the tracer in both sexes. After completion of this study, permission was received to include 9 patients with prostate cancer to evaluate tumor uptake kinetics (22).

## MATERIALS AND METHODS

The study (NCT03995888) was authorized by the Finnish Medicines Agency. Ethical approval was received from the Ethics Committee of the Hospital District of Southwest Finland, and all subjects gave written informed consent. The study was conducted in accordance with good clinical practice guidelines.

### Subjects

Six healthy adult volunteers (3 men, 3 women) meeting the following criteria were enrolled between June 18 and August 14, 2019: an age of 21–65 y, the ability to provide informed written consent, a body mass index of less than 30 kg/m^2^ and body weight of less than 90 kg, negative test results for drugs of abuse and alcohol, willingness to abstain from sexual intercourse for 24 h after ^18^F-rhPSMA-7.3 administration, and willingness to practice effective contraception for 3 mo after ^18^F-rhPSMA-7.3 administration (men) or having a postmenopausal or surgically sterile status (women). Exclusion criteria included participating in another clinical trial in the 3 mo before planned administration of ^18^F-rhPSMA-7.3, being significantly exposed to ionizing radiation in the preceding 12 mo, undergoing monitoring for occupational ionizing radiation exposure, or having claustrophobia, bilateral hip prostheses, or positive test result for hepatitis B, hepatitis C, or HIV.

### Radiopharmaceutical Preparation

^18^F-rhPSMA-7.3 was produced on site at Turku PET Centre using a single-use cassette-based proprietary automated synthesis platform for radiolabeling, purification, and formulation (Scintomics GRP; Scintomics GmbH) and using an in-house remotely operated sterile filtration device for aseptic filling, in accordance with good manufacturing practices and Turku PET Centre’s standard procedures.

### Subject Preparation and ^18^F-rhPSMA-7.3 Administration

Subjects were requested not to eat for at least 4 h before the administration of ^18^F-rhPSMA-7.3 and to remain well hydrated before the scan. Subjects were encouraged to void immediately before ^18^F-rhPSMA-7.3 injection. A venous cannula was placed in each arm. ^18^F-rhPSMA-7.3 (target radioactive dose, 225 MBq ± 10%) was administered as an intravenous bolus injection, followed by a flush with 5 mL of saline solution. The viability of the cannula for blood samples was ensured by actively infusing saline (≤500 mL) through an intravenous drip for the duration of the scan.

### Image Acquisition and Reconstruction

**FIGURE 1.**
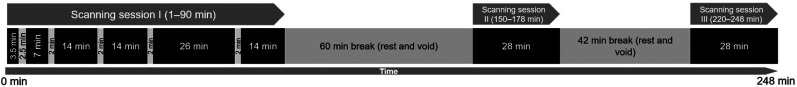
^18^F-rhPSMA-7.3 PET schedule. Scans and their total duration are represented as black boxes, and breaks are represented as gray boxes. All scans were conducted from vertex to mid thigh, apart from scan 5 (26 min), which was conducted from vertex to feet.

All images were captured using a Discovery MI PET/CT scanner (GE Healthcare). The subjects underwent 3 low-dose CT scans for attenuation correction and anatomic correlation, each followed by multiple whole-body PET acquisitions at scheduled times up to 248 min after injection (Fig. 1). PET scans were conducted in 3 separate sessions as outlined in Figure 1 and Supplemental Table 1 (supplemental materials are available at http://jnm.snmjournals.org). The PET images were reconstructed using a 3-dimensional iterative algorithm (VuePoint Fx; GE Healthcare) with 4 iterations and 8 subsets and using a standard *z*-axis filter with a 7.0-mm filter cutoff.

### Safety Assessments

Any adverse event that occurred from the time of informed consent throughout the study period was recorded. Laboratory parameters (serum biochemistry, hematology, coagulation, and urinalysis) were monitored during the 24 h after ^18^F-rhPSMA-7.3 administration. Venous blood samples were collected at baseline and at 90, 180, and 250 min after injection, with a further sample collected approximately 24 h after injection. Urine samples were collected at baseline and at 250 min and 24 h after injection. A standard physical examination was performed during the screening visit, with further brief examinations performed before the administration of ^18^F-rhPSMA-7.3 and again at discharge from the site. A 12-lead electrocardiogram was recorded during the screening visit, twice at baseline before injection (at 120–15 min and again at 5 min), 3 times after injection (at 90, 180, and 250 min), and again at approximately 24 h. Resting vital signs (body temperature, respiration rate, supine systolic and diastolic blood pressure, and heart rate) were measured during the screening visit; at baseline 5 min before injection; at 2, 5, 10, 15, 30, 60, 90, 180, and 250 min after injection; and at approximately 24 h after injection. The cannulation sites were checked regularly for signs of any adverse effects.

### Assessment of Pharmacokinetics

Blood and urine samples were collected to assess ^18^F radioactivity in whole blood, plasma, and urine. Blood samples for ^18^F radioactivity analysis were collected via a peripheral venous cannula, and the analysis was conducted with an automatic γ-counter (Wizard 1480 3″; Wallac). Blood samples (2 mL) were collected at approximately 30 s, 60 s, 90 s, 4½ min, 5 min, 6 min, 7 min, 8 min, 15 min, 31 min, 47 min, 75 min, 120 min, 180 min, and 250 min after injection.

Urine samples were collected before injection of ^18^F-rhPSMA-7, between scanning sessions 1 and 2, between scanning sessions 2 and 3, and after scanning session 3 (approximate timings: up to 5 min before injection, from 5 min before injection to 95 min after injection, from 95 to 185 min after injection, and from 185 to 255 min after injection, respectively).

### Biodistribution and Radiation Dosimetry

Quantitative measurements of ^18^F radioactivity in volumes of interest (VOIs) over target organs captured in whole-body images were made at 8 postinjection time points. The target areas comprised muscle, liver, lungs, cardiac wall, cardiac chamber content, kidneys, brain, breasts (women only), spleen, stomach, urinary bladder content, thymus, cortical bone, trabecular bone, parotid gland, submandibular salivary gland, sublingual salivary gland, lacrimal gland, upper and lower large intestine content, small intestine content, uterus (women only), pancreas, thyroid, red marrow, gallbladder content, adrenals, and testes (men only).

Time–activity concentration curves were generated and were normalized to a 1-MBq injected dose and to the organ weights of a 70-kg reference man. Normalized curves were fitted with an exponential function until infinity in Microsoft Excel, version 2013. In cases of continuing uptake in the source organ, only the descending part of the curve was used. The area under the time–activity concentration curves was used to determine the cumulated activities in the source organs.

Two methods were used to account for the radioactivity in the urinary bladder. First, the cumulated activity in the urinary bladder content was calculated using VOIs and the volume of voided urine. The volume of urinary bladder content was first measured after scanning session 1 at 111 min (range, 103–126 min), and this value was multiplied by the urinary bladder content VOI values determined from the 6 scans in session 1. The second volume measurement was taken at 194 min (range, 190–198 min) and multiplied by the VOI value from scan 7. The third volume measurement (263 min; range, 257–271 min) was multiplied by the VOI value from scan 8. The second approach used the dynamic bladder model (23), which estimates the biologic half-life from the urine samples on the assumption that there is no other route of ^18^F-rhPSMA-7.3 excretion. The data from the dynamic bladder model were used in the subsequent dosimetry analysis. We modeled for both 1-h and 3.5-h voiding intervals with this method.

For internal radiation dosimetry calculations, the cumulated tissue radioactivity estimates for each subject were fed into the OLINDA/EXM program, version 1.0, which makes use of the MIRD schema (24,25). Absorbed doses in MIRD-specified target regions were estimated using the Cristy–Eckerman 70-kg adult male phantom (26–28), and the subjects’ effective dose was calculated from these absorbed dose data. Organ mass values used in the OLINDA/EXM program were determined from the Cristy–Eckerman model but with the addition of further source organs in which PSMA uptake is known to be relatively high: parotid glands (25 g), sublingual salivary glands (12.5 g), submandibular salivary glands (12.5 g), and lacrimal glands (5 g).

### Statistics

Statistics in this dosimetry study were limited to descriptive statistics, that is, mean values for the calculated doses and their SD from the mean.

## RESULTS

The 6 participants had a mean age of 52 y (range, 25–64 y) and a mean body mass index of 26.1 kg/m^2^ (range, 23.2–29.7 kg/m^2^).

### Safety

Five treatment-emergent adverse events were reported. Four of these (a focal liver lesion on a scan [MRI-confirmed normal liver], dizziness, headache, and sinusitis) were mild or moderate and were judged not to be associated with ^18^F-rhPSMA-7.3. One adverse event was judged to be possibly associated with ^18^F-rhPSMA-7.3. The subject reported a mild headache that started after discharge from the site, 6 h after the injection, and continued until the next morning (duration, 14 h). The subject’s 24-h laboratory results were within reference ranges, and the physical examination, vital signs, and electrocardiogram showed no changes from previous findings. No medication was needed to treat this adverse event. Because of the temporal association with ^18^F-rhPSMA-7.3 injection, a causal relationship could not be excluded.

### Biodistribution

**FIGURE 2.**
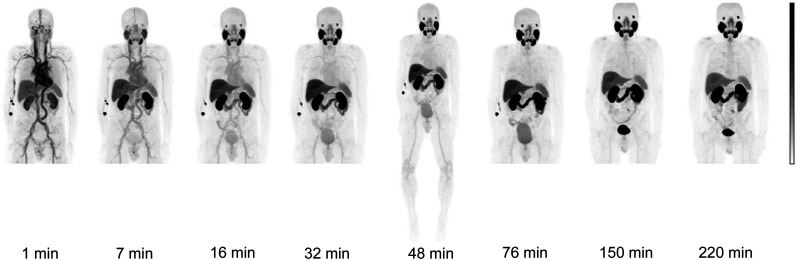
PET images of representative healthy volunteer after administration of ^18^F-rhPSMA-7.3. First scan session was at 1–90 min after injection, second was at 150–178 min, and third was at 220–248 min. Subject was permitted to leave PET scanner and void urine between sessions.

**FIGURE 3.**
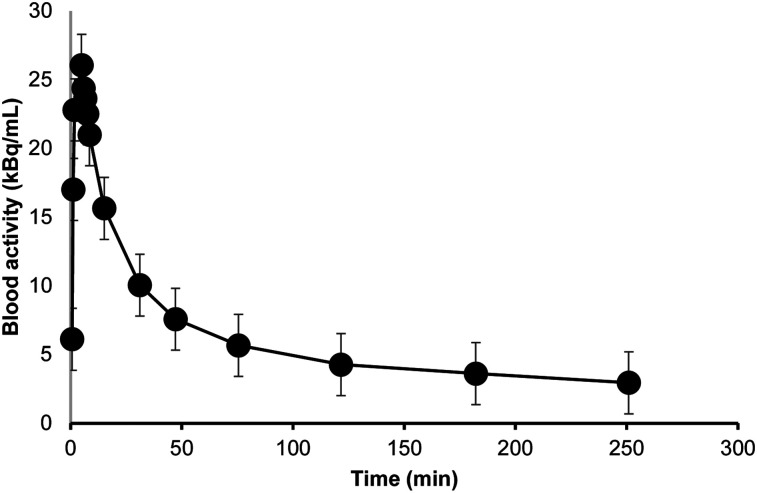
Radioactivity concentration of ^18^F-rhPSMA-7.3 in whole blood as function of time. *x*-axis values represent mean time of sample collection, and *y*-axis values represent mean activity, from all subjects.

Figure 2 presents PET images of a representative subject after the administration of ^18^F-rhPSMA-7.3. The mean decreasing decay-corrected concentration of ^18^F-rhPSMA-7.3 in whole blood as a function of time is presented in Figure 3. The curve presents data from all subjects, and we observed no significant differences in the derived concentration curves between male and female subjects.

The subjects exhibited high initial (1 min after injection) ^18^F uptake in the liver (mean proportion of injected radioactivity, 15.8%; range, 13.9%–17.0%), heart content (mean proportion of injected activity, 7.4%; range, 6.5%–9.2%), and cortical bone (mean proportion of injected activity, 3.5%; range, 3.0%–4.4%). Skeletal muscle also showed relatively high initial uptake as a consequence of its large share of total-body volume (mean proportion of injected activity, 24.3%; range, 19.2%–29.3%). The brain and pancreas showed little initial uptake (mean proportion of injected radioactivity, 0.8% for brain [range, 0.6%–1.1%] and 0.6% for pancreas [range, 0.4%–0.9%]).

**FIGURE 4.**
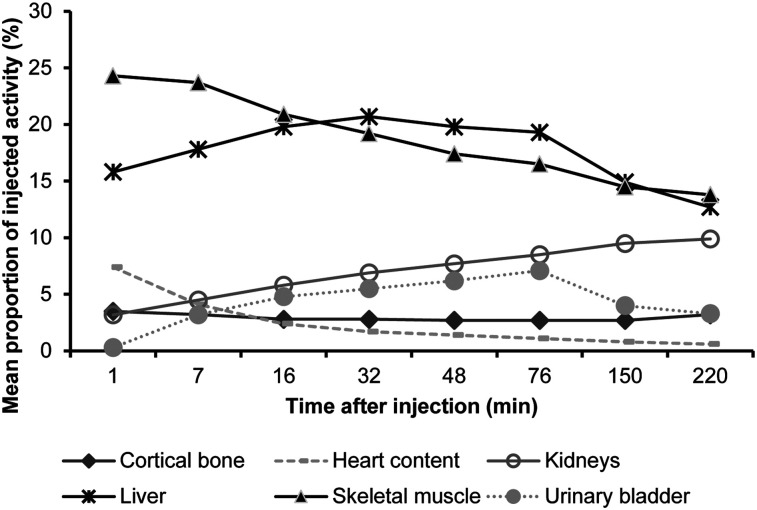
Organ-specific relative uptake (top 6 organs).

Over the whole scan period, the organs with the highest relative uptake were skeletal muscle, the liver, and the kidneys (Fig. 4). The low initial ^18^F activity in the brain and pancreas decreased throughout the scanning period. The testes and gallbladder showed almost no uptake throughout the scans. The thymus and thyroid each accounted for only 0.1% of the injected radioactivity during the first few minutes, and activity subsequently fell to zero. The lacrimal glands showed no uptake until 76 min after injection, whereupon 0.1% of the injected activity could be detected. Several organs (the adrenals, brain, breasts, upper and lower large intestines, pancreas, parotid gland, stomach, sublingual gland, submandibular gland, trabecular bone, and uterus) showed a mean uptake of less than 1% of injected radioactivity for the entire scan period. The relative uptake in the lungs, red marrow, small intestine, spleen, and heart wall was less than 3% throughout the scan period, with all but the spleen and red marrow showing decreasing activities over the scanning period.

At the end of the first scanning session (mean time, 111 min after injection), the mean results from the 6 participants indicated that 7.2% (range, 4.4%–9.0%) of the injected radioactivity of ^18^F-rhPSMA-7.3 was excreted into urine. The mean values for measured activities in urine samples and the relative proportion of injected activity in the urine are shown in Supplemental Table 2. The data reveal that the mean cumulative proportion of ^18^F-rhPSMA in urine was 7.2%, 11.4%, and 14.8% after scanning sessions 1, 2, and 3, respectively.

### Radiation Dosimetry

Biodistribution data from the 6 participants were used to calculate organ-specific and effective doses using the adult male phantom. The calculated effective dose was 0.0138 mSv/MBq when a 1-h voiding interval was used and 0.0141 mSv/MBq with a 3.5-h voiding interval (Table 1). All organs were found to have the same absorbed doses when modeled with a 1-h or a 3.5-h voiding interval, with the exception of the urinary bladder wall, which had a mean absorbed dose of 0.006 ± 0.001 mGy/MBq with a 1-h interval and 0.012 ± 0.003 mGy/MBq with a 3.5-h interval. The organs with the highest mean absorbed doses per unit of administered radioactivity were the adrenals (0.184 mGy/MBq), the kidneys (0.172 mGy/MBq), and the submandibular glands (0.148 mGy/MBq) (Table 1). Individual patient data are available in Supplemental Table 3.

**TABLE 1:** Mean Organ-Specific Absorbed Doses and Effective Dose Calculated Using Cristy–Eckerman Adult Male Phantom and 3.5-Hour Voiding Interval

Organ	Mean absorbed dose* (mGy/MBq)	SD
Adrenals	0.184	0.053
Kidneys	0.172	0.030
Submandibular glands	0.148	0.043
Parotid glands	0.114	0.025
Spleen	0.083	0.024
Lacrimal glands	0.080	0.038
Sublingual glands	0.065	0.036
Liver	0.062	0.006
Pancreas	0.028	0.005
Heart wall	0.020	0.003
Gallbladder wall	0.017	0.001
Urinary bladder wall	0.012	0.003
Stomach wall	0.012	0.001
Small intestine	0.012	0.003
Osteogenic cells	0.012	0.002
Uterus	0.011	0.008
Thymus	0.010	0.001
Upper large intestine wall	0.010	0.001
Lungs	0.010	0.001
Red marrow	0.010	0.002
Thyroid	0.010	0.002
Lower large intestine wall	0.007	0.002
Muscle	0.006	0.001
Testes	0.005	0.003
Ovaries	0.005	0.001
Breasts	0.004	0.002
Skin	0.002	0.000
Brain	0.002	0.000
Mean effective dose (mSv/MBq)*	0.014	0.001

* Mean dose from all 6 subjects.

## DISCUSSION

^18^F-rhPSMA-7.3 is a promising novel PET radiopharmaceutical for the imaging of PSMA, which is upregulated in prostate cancer cells. Here, we evaluated the clinical safety, biodistribution, and internal radiation dosimetry of ^18^F-rhPSMA-7.3 in 6 healthy adult volunteers. ^18^F-rhPSMA-7.3 was found to be well tolerated, with all subjects showing normal laboratory parameters throughout. Five treatment-emergent adverse events occurred in 2 subjects. Only one of these events (headache) was considered possibly related to ^18^F-rhPSMA-7.3 administration.

The mean effective dose of ^18^F-rhPSMA-7.3, 0.0141 mSv/MBq, appears favorable and lower than the reported effective doses of other established PSMA ligands, such as ^18^F-DCFPyL (0.0165 mSv/MBq) (29), ^68^Ga-PSMA-11 (0.0158 mSv/MBq) (30), and ^18^F-PSMA-1007 (0.0220 mSv/MBq) (9). An injection of 300 MBq of ^18^F-rhPSMA-7.3 would result in an effective dose of approximately 4.2 mSv. This is relatively low compared with common imaging procedures and therefore potentially allows the use of ^18^F-rhPSMA-7.3 for repeated PET scans, such as for therapeutic follow-up of patients with prostate cancer (31).

The present study represents a genuine phase 1 safety and dosimetry study. It has always been common to perform (ethics-approved) radiation dosimetry studies of novel PET imaging agents in healthy volunteers before initiation of clinical trials in patients. Since the introduction of PSMA ligands, however, clinical use of these tracers has preceded the dosimetric and safety analyses. It is worth noting that only one other study with a PSMA PET tracer was conducted in healthy volunteers (9), whereas other evaluations were conducted in patients (29,30). Given the uptake of PSMA ligands in tumors, we believe that patient studies do not provide the best representation of a normal biodistribution. The value of determining the normal biodistribution of new radiopharmaceuticals should not be underestimated.

Furthermore, this study followed the classic design, establishing safety and dosimetry in both male and female volunteers. All other PSMA dosimetry studies have been conducted only in men. Given the fact that PSMA ligands can be used to image diseases other than prostate cancer (32–34), their safety and biodistribution in women have to be established for regulatory purposes if one might conduct studies or seek market authorization for other indications. The data obtained in men and women can then safely be used in the adult male model described by Cristy and Eckerman, since it is hermaphroditic and could also represent a larger-than-average—that is, more than 58 kg—adult female (28).

The extent of excretion of PSMA ligands in urine is a highly relevant product characteristic, given its potential interference with the interpretability of prostate bed scans. Our data suggest that ^18^F-rhPSMA-7.3 is also cleared via the urinary system; excretion of ^18^F-rhPSMA-7.3 into the bladder was notable from the second scan (7 min after injection) and further increased throughout the scanning period. Activity in the urinary bladder was still visible during the second and third scanning sessions despite the subjects’ voiding during the intersession breaks and the decreasing excretion via the urine over time. Considerable intersubject variation was observed in urinary excretion. The average excretion of ^18^F-rhPSMA-7.3 in the urine was measured to be 7.2% (range, 4.4%–9.0%). That is less than the average urinary excretion of ^18^F-DCFPyL (11%) (29) and ^68^Ga-PSMA-11 (11%) (30) in the first 2 h but more than the average urinary excretion of ^18^F-PSMA-1007 in the first 2 h (1.2%) (9), since this compound is mainly excreted via bile.

To fully determine the potential of a new PET tracer and establish its usefulness for detecting disease in normal organs, assessing the extent of physiologic uptake in these organs is essential. In general, the biodistribution of ^18^F-rhPSMA-7.3 was found to be similar to that of other PSMA-based tracers, typically showing high uptake in salivary glands and the kidneys, and in line with the known expression of PSMA in these healthy tissues (1,35). Although possible binding to PSMA present in the kidneys may not negatively affect prostate imaging, it may interfere with the detection of primary tumors of the kidney. Studies have shown that PSMA ligands can detect metastases of renal cell carcinoma (32,33) but that the primary tumors could not be visualized with the PSMA tracer (33). Furthermore, high uptake in and hence high absorbed doses to the kidneys may have a negative impact on therapeutic use of PSMA ligands labeled with β- or α-emitters. The same holds true for the high uptake of ^18^F-rhPSMA-7.3 in the salivary glands. Although this uptake does not interfere with the interpretability of the PET scan, absorbed doses from β- or α-emitters bound to PSMA ligands can cause serious harm (36,37).

The organ with the highest mean absorbed doses per unit of administered radioactivity was the adrenals. These doses seem higher than the doses reported by other studies, but the other studies did not draw VOIs around the adrenals (*9,30*). That fact implies that the other studies assumed radiation to the target, the adrenal glands, only from other adjacent source organs (e.g., the kidneys) and not from the adrenals themselves. When assuming that the adrenals are also a source organ for their own absorbed dose, the absorbed dose evidently rises. Furthermore, interindividual variation in the absorbed doses was largest in the adrenals, along with other small organs such as the sublingual, lacrimal, and submandibular glands. These were the smallest organs that were analyzed, and standardized organ weights were attributed to these glands. Interindividual variation of the actual organ weight may have contributed to this variation in calculated doses. Furthermore, it is possible that small mismatches in aligning the discrete VOIs and the actual boundaries of the organs, as well as possible partial-volume effects, may have increased this variation.

Potential limitations of the present work include the use of standardized organ weights as discussed above and the small number of study subjects, although a small number of subjects is standard for studies of this nature.

## CONCLUSION

The present data acquired in healthy subjects indicate that ^18^F-rhPSMA-7.3 is a well-tolerated PET radiopharmaceutical with a favorable radiation dosimetry profile and an effective dose that is suitable for clinical imaging.

## DISCLOSURE

This study was funded by Blue Earth Diagnostics Ltd. (BED), Oxford, U.K. Tuula Tolvanen, Salla Lahdenpohja, and Ernst Postema received personal fees from BED during the conduct of this study. Mika Scheinin is an employee, shareholder, and board member of CRST Ltd. Mika Scheinin, Simona Malaspina, Anna Kuisma, and Kari Kalliokoski received funding from BED for contract research in relation to this study. Matthew Miller is an employee and shareholder of BED. No other potential conflict of interest relevant to this article was reported.

**KEY POINTS**

**QUESTION:** Are the biodistribution, internal radiation dosimetry, and safety profile of ^18^F-rhPSMA-7.3 suitable for PET imaging?
**PERTINENT FINDINGS:** For a mean administered activity of 220 MBq, there were no adverse or clinically detectable pharmacologic effects in any of the 6 subjects, who were imaged at multiple time points over a 4-h period. No significant changes were observed in vital signs or in the results of laboratory studies or electrocardiograms. The mean effective dose (0.0141 mSv/MBq) was favorable and lower than that of many established PSMA ligands.
**IMPLICATIONS FOR PATIENT CARE:** The biodistribution and radiation dosimetry of ^18^F-rhPSMA-7.3 are favorable for PET imaging; ^18^F-rhPSMA-7.3 shows potential for safe use, even for repeated scans as might occur in therapeutic follow-up of prostate cancer.
